# A Case of Venous Thoracic Outlet Syndrome: Primary Care Review of Physical Exam Provocative Tests and Osteopathic Manipulative Technique Considerations

**DOI:** 10.7759/cureus.4921

**Published:** 2019-06-17

**Authors:** Eric Yuschak, Furqan Haq, Stacy Chase

**Affiliations:** 1 Family Medicine, St. Petersburg General Hospital, St. Petersburg, USA; 2 Internal Medicine, Oak Hill Hospital, Tampa, USA

**Keywords:** thoracic outlet syndrome, osteopathic manipulative treatment, physical exam skills

## Abstract

Thoracic outlet syndrome (TOS) is a condition that results from the compression of the neurovascular bundle of the upper extremities. It has generally been divided into neurogenic and vascular TOS with vascular further being divided into venous and arterial. This case report is of a healthy 24-year-old female who was misdiagnosed initially in an emergency department only to return two days later for reevaluation. Here, the patient's admission and workup are discussed after she presented to the family medicine clinic for a referral for further evaluation by a cardiothoracic surgeon. Physical exam provocative tests which are often overlooked are reviewed, and osteopathic manipulative techniques and principles are mentioned.

## Introduction

Anatomically, the thoracic outlet is the area containing the brachial plexus, subclavian artery, and subclavian vein as it passes from the neck into the upper extremity [1]. The bony structures include the spinal column, first rib, and sternum. The two most common areas of impingement leading to thoracic outlet syndrome include the scalene triangle and the costoclavicular space. The scalene triangle is most common for brachial plexus compression, leading to neurogenic thoracic outlet syndrome (TOS) as the subclavian vein courses anteromedial to this space. The borders include the anterior scalene muscle anteriorly, middle scalene posteriorly, and superior border of the first rib inferiorly. The costoclavicular space is the most common area leading to vascular TOS and is between the first rib and clavicle. A third of the area of compression that is not considered the thoracic outlet is the pectoralis minor space between the pectoralis minor muscle anteriorly and chest wall posteriorly. Compression is commonly caused by either congenital or acquired anatomic anomalies. Congenital anomalies include cervical ribs, supernumerary muscles, abnormal tendon insertions, and abnormal muscular or tendinous bands. Acquired anomalies include bony callus after fracture and muscle hypertrophy from repetitive movements such as lifting.

Neurogenic TOS comprises approximately 95% of cases while venous TOS is the second-most common with about 4% of cases [2]. Neurogenic versus vascular TOS cases are distinguished by clinical presentation and distinct site of compression as discussed, however, symptoms can overlap. Typically, neurogenic TOS presents with numbness and weakness secondary to the brachial plexus compression while venous TOS results from compression of the venous return of the upper extremity leading to extremity swelling, cyanosis, and possibly deep venous thrombosis (DVT).

The deep-venous thrombosis is secondary to stasis of the blood or occlusion of the vessel which is part of Virchow’s Triad along with endothelial injury and hypercoagulability. This is considered a spontaneous upper-extremity DVT which has also been termed Paget Schroetter syndrome or an “effort” thrombosis [3-4]. It is defined as a thrombus of the deep veins draining the upper extremity due to anatomic abnormalities of the thoracic outlet, causing axillosubclavian compression and subsequent thrombosis. The symptoms again include extremity swelling, cyanosis, heavy feeling, a palpable venous cord and possible neurologic symptoms such as weakness and numbness from compression secondary to swelling. Risk factors include younger age, athletic muscular males, strenuous upper extremity activity, repetitive overarm hyperabduction, anatomic abnormalities of the thoracic outlet, and thrombophilia. A workup for a spontaneous upper-extremity DVT includes a thorough history and physical, imaging, and screening for hypercoagulability. History and physical is aimed at the onset of symptoms and personal and family history of previous blood clots. Imaging typically utilizes Doppler ultrasound to check for venous outflow obstruction and venous compressibility. Finally, hypercoagulability screening is completed. This case report specifically focuses on the case of a young healthy adult female who was diagnosed with venous TOS.

## Case presentation

A 24-year-old Caucasian female presented to the emergency department (ED) with the chief complaint of right arm pain for the past four days. The pain started in her neck and radiated to the right trapezius and down to her elbow. She said she was seen at a different ED two days prior and was treated for a pinched nerve by being given dexamethasone, cyclobenzaprine, and ibuprofen. However, the pain worsened and she noticed color changes of her arm that were dependent on position. She said her hand was turning blue unless she held it above her head. She had a past medical history significant only for menstrual migraines and had been on oral contraception since she was a teenager. She denied tobacco or recreational drug use and drinks alcohol socially. She exercises regularly but the type of exercise was not specified. She admitted to a family history of cervical cancer in her mother, a grandfather with polycythemia and a grandmother with diabetes, hypertension and biliary duct cancer. Her recorded allergies included penicillin, latex, and adhesive tape.

Her workup in the ED included labs and imaging. The labs appeared normal except for a leukocytosis of 14.4 k/mm^3^ and neutrophils of 12.1 k/mm^3^. She had a chest X-ray without acute cardiopulmonary process. A venous Doppler ultrasound was reported as significant for an acute right axillary/subclavian deep venous thrombosis (DVT). She was admitted and seen by vascular surgery, cardiology, and hematology. She underwent catheter-directed thrombolysis and was started on anticoagulation therapy. She had a hypercoagulability workup which included phosphatidylserine/prothrombin antibody IgG/IgM, factor 5 mutation, antithrombin 3, antinuclear antibody, rheumatoid factor, factor 3 mutation, anti-Xa heparin, protein C, and protein S, all of which was negative. She was discharged on warfarin after a therapeutic international normalized ratio (INR) was achieved. Obstetrics and gynecology recommended discontinuing the oral contraceptive pills. She continued to follow up with her primary care physician and hematologist who was managing her warfarin and the diagnosis of venous thoracic outlet syndrome was made. She now presented to the office after moving to the area and requested a referral to cardiothoracic surgery to complete her evaluation for possible thoracic outlet decompression surgery.

## Discussion

**Figure 1:**
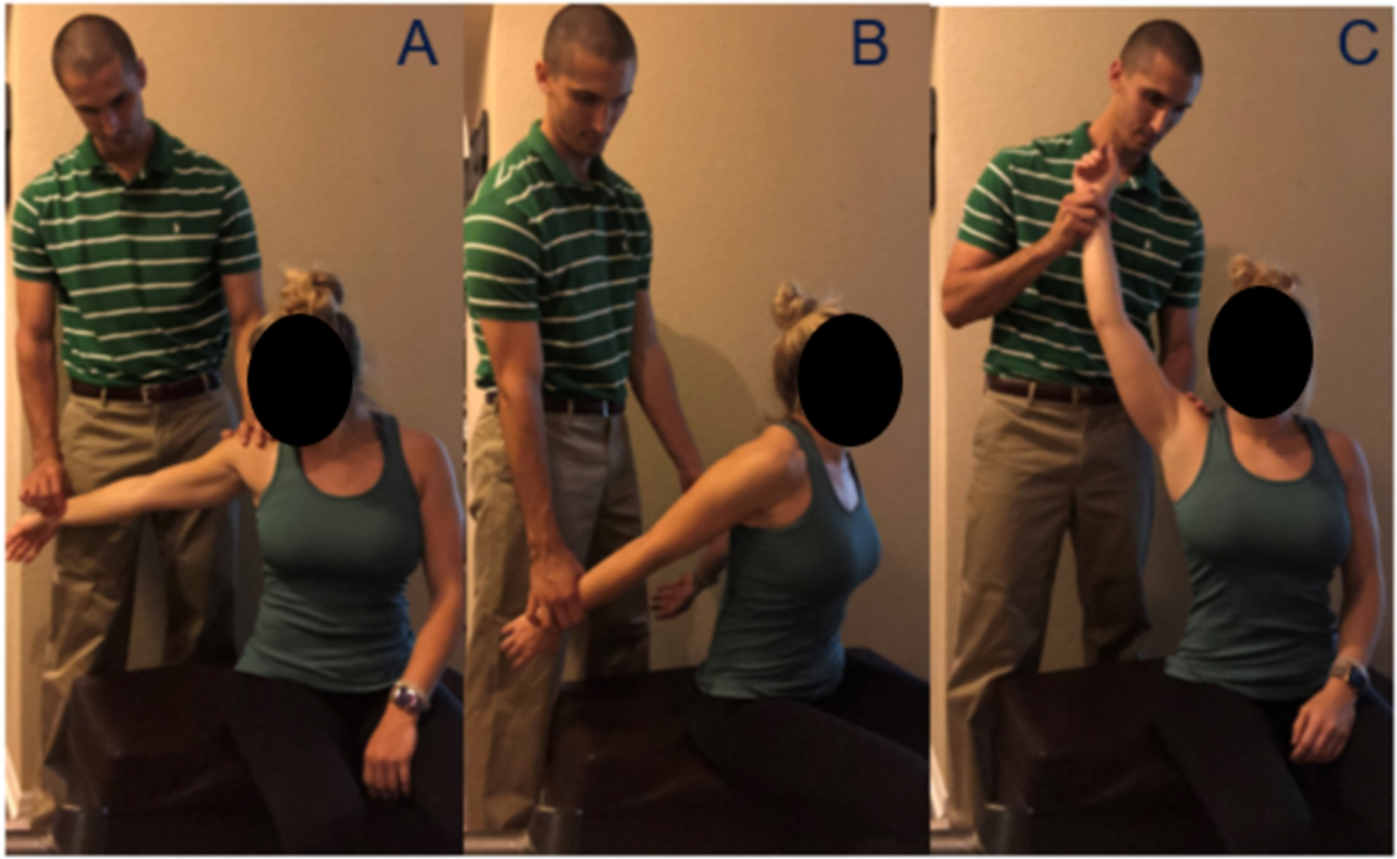
Physical Exam Provocative Maneuvers Panel A: Adson's Test Panel B: Costoclavicular Test/Military Posture Panel C: Wright's Test/Hyperabduction

Initial physical exams are important as they can help identify the area of compression. Adson’s test indicates compression between the scalene muscles; the costoclavicular test, also known as the military posture test, indicates compression between the clavicle and first rib; and the hyperabduction test, also known as Wright’s test, indicates compression between the pectoralis minor and chest wall [5]. For each test the radial pulse is monitored by the physician and a positive test can be from loss of pulse, paresthesia, or pain in the manipulated arm. Figure 1 demonstrates the three different tests. The Adson’s test (Figure 1, Panel A) for scalene syndrome is performed by extending the patients arm at the elbow and extending, externally rotating and slightly abducting the shoulder. The radial pulse is monitored and the patient is asked to take a deep breath and turn their head to the ipsilateral side. The costoclavicular test (Figure 1, Panel B) for costoclavicular syndrome is performed by depressing and extending the shoulder while monitoring the radial pulse. The patient adopts a “military posture” with shoulders back and down and the chest out. Finally, the hyperabduction test (Figure 1, Panel C) is performed by hyperabducting the arm above the head with some extension. Venous TOS is more commonly caused by compression in the costoclavicular space making military posture test most likely to be positive.

The treatment of neurogenic and vascular TOS differ [6]. Neurogenic TOS more commonly utilizes stretches, therapy for strengthening, and manipulations to decrease muscle hypertonicity and postural imbalances. However, venous TOS treatment, especially at the stage of thrombosis, includes a minimum of three months of anticoagulation as recommended for the American College of Chest Physicians, thrombolysis if less than two weeks duration, and possibly thoracic outlet decompression.

Osteopathic manipulations have also been shown to improve symptoms of thoracic outlet syndrome and are more commonly utilized in neurogenic TOS. Targeted areas include the cervical spine levels C2-C7, the thoracic spine level T1, rib 1, thoracic inlet, clavicle, and scalenes if somatic dysfunction is present [7]. A paper by Dobrusin comments on how dysfunctional area treatment can, "decrease muscular tone in the scalene muscles, allow the first rib to become more mobile, and open up the interscalene triangle" [8]. A combination of high velocity/low amplitude, counterstrain, and myofascial release osteopathic manipulative techniques can be used based on patient presentation and clinician experience. In addition Still’s technique, which is an articulatory technique that incorporates indirect and direct techniques, can be effective.

## Conclusions

This case reviewed a less common presentation of thoracic outlet syndrome. It also demonstrates the importance of physical exam. While there is often pressure to see many patients in a short amount of time in addition to proper documentation it is important not to forget the basics with history and a thorough physical exam. A simple review of physical exam provocative tests may have helped make a correct diagnosis upon initial evaluation. Additionally, as an osteopathic family medicine resident, understanding additional osteopathic manipulative treatment techniques that may benefit a patient is useful, and continual practice is necessary to develop and refine the skill of manipulation. 
